# Systematic review of prediction models for gestational hypertension and preeclampsia

**DOI:** 10.1371/journal.pone.0230955

**Published:** 2020-04-21

**Authors:** Edward Antwi, Mary Amoakoh-Coleman, Dorice L. Vieira, Shreya Madhavaram, Kwadwo A. Koram, Diederick E. Grobbee, Irene A. Agyepong, Kerstin Klipstein-Grobusch

**Affiliations:** 1 Julius Global Health, Julius Center for Health Sciences and Primary Care, University Medical Center Utrecht, Utrecht University, Utrecht, The Netherlands; 2 Ghana Health Service, Accra, Ghana; 3 Epidemiology Department, Noguchi Memorial Institute for Medical Research, College of Health Sciences, University of Ghana, Legon, Accra, Ghana; 4 New York University Health Sciences Library, New York University School of Medicine, New York, NY, United States of America; 5 Division of Epidemiology & Biostatistics, School of Public Health, Faculty of Health Sciences, University of the Witwatersrand, Johannesburg, South Africa; University of Cambridge, UNITED KINGDOM

## Abstract

**Introduction:**

Prediction models for gestational hypertension and preeclampsia have been developed with data and assumptions from developed countries. Their suitability and application for low resource settings have not been tested. This review aimed to identify and assess the methodological quality of prediction models for gestational hypertension and pre-eclampsia with reference to their application in low resource settings.

**Methods:**

Using combinations of keywords for gestational hypertension, preeclampsia and prediction models seven databases were searched to identify prediction models developed with maternal data obtained before 20 weeks of pregnancy and including at least three predictors (Prospero registration CRD 42017078786). Prediction model characteristics and performance measures were extracted using the CHARMS, STROBE and TRIPOD checklists. The National Institute of Health quality assessment tools for observational cohort and cross-sectional studies were used for study quality appraisal.

**Results:**

We retrieved 8,309 articles out of which 40 articles were eligible for review. Seventy-seven percent of all the prediction models combined biomarkers with maternal clinical characteristics. Biomarkers used as predictors in most models were pregnancy associated plasma protein-A (PAPP-A) and placental growth factor (PlGF). Only five studies were conducted in a low-and middle income country.

**Conclusions:**

Most of the studies evaluated did not completely follow the CHARMS, TRIPOD and STROBE guidelines in prediction model development and reporting. Adherence to these guidelines will improve prediction modelling studies and subsequent application of prediction models in clinical practice. Prediction models using maternal characteristics, with good discrimination and calibration, should be externally validated for use in low and middle income countries where biomarker assays are not routinely available.

## Introduction

Hypertensive disorders of pregnancy (HDPs) are important causes of maternal morbidity and mortality globally but the burden is greatest in low- and middle-income countries (LMIC) [1–3]. These disorders of pregnancy include gestational hypertension, preeclampsia and eclampsia and are characterized by an increase in blood pressure and multi-organ derangements which range from mild to severe [4]. There is no known cure but daily administration of low dose aspirin early in the first trimester has been shown to reduce the incidence and the severity of preeclampsia [5–8]. Preeclampsia is a major indication for preterm delivery, accounting for about 15% of all preterm deliveries [9–13] and is a cause of increased healthcare costs through the prolonged stay of the mother or newborn in intensive care units [14].

Prediction models provide estimates of the probability or risk of the future occurrence of a particular outcome or event in individuals at risk of such an event [15]. Prediction models have also been used to identify women at high risk of developing HDPs later in pregnancy so as to provide for closer monitoring from early pregnancy onwards, including low dose aspirin prophylaxis [5–8] which has been shown to reduce the risk of developing preeclampsia.

The aim of this systematic review was to evaluate the performance of multivariate prediction models to address the question of the effectiveness of prediction models in identifying pregnant women at risk of gestational hypertension and preeclampsia. The objectives were to identify prediction models for gestational hypertension and preeclampsia; assess the methodological quality of the studies to develop and externally validate the prediction models using the CHARMS [16] checklist; and to identify prediction models that can be applied in low and middle income country settings.

## Methods

This study was conducted using the critical appraisal and data extraction for systematic reviews of prediction modelling studies (CHARMS) [16], strengthening the reporting of observational studies in epidemiology (STROBE) [17] and the transparent reporting of a multivariable prediction model for individual prognosis or diagnosis (TRIPOD) [18] checklists. The Population, Intervention, Comparator and Outcome (PICO) format for the review was as follows: P (pregnant women), I (prediction models), C (none) and O (gestational hypertension or preeclampsia). The study protocol was registered with the Prospero International Prospective Register of Systematic Reviews (CRD 42017078786).

### Search strategy

A comprehensive systematic literature search with was conducted in PubMed/Medline, Embase, Cochrane Library, Web of Science and CINAHL databases from their inception through 18 September 2017. The search was updated to 15 October 2019 (DLV,EA). The MeSH database, EMTREE subject headings and CINAHL subject headings were used to construct the search strategy along with author keywords and general keywords. In addition, an electronic hand search was conducted in a number of journals from 10th September through 25^th^ September, 2017 and from October 1 to October 15, 2019. Finally, grey literature was searched using the New York Academy of Medicine Grey Literature, OCLC’s OAISTER, and Open Grey databases.

The search strategy is provided as a supplementary file (S1 Data).

### Eligibility/Inclusion criteria

Cohort studies, nested-case control studies and randomized controlled trials were eligible for inclusion in the study. Case-control, cross-sectional, animal studies, bio-molecular studies, letters, reviews and case reports were excluded because for prediction modeling studies we require absolute risks whereas case-control or cross-sectional studies only give relative risks. The primary outcomes for the included studies were gestational hypertension and preeclampsia.

### Definition of terms

Gestational hypertension was defined as elevated systolic blood pressure equal to or greater than 140mmHg and/or diastolic blood pressure equal or greater than 90mmHg on at least two occasions four hours apart and appearing for the first time after 20 weeks of gestation without proteinuria [4]. Pre-eclampsia was defined as gestational hypertension with proteinuria of 300mg or more in a 24-hour urine sample or spot urine protein/creatinine ratio of 30mg/mmol [4]. Pre-eclampsia was further divided into early-onset preeclampsia (requiring preterm delivery before 34 weeks gestation) and late-onset preeclampsia (with delivery at or after 34 weeks gestation or later) as an outcome by some studies [19–24].

A prediction model [25] was defined as a logistic regression formula or a survival model with three or more predictors that could be used to estimate risk probabilities for individual patients or to distinguish between groups of patients of different risks.

### Screening methods for study identification

Two reviewers (EA, MAC) independently assessed the titles and abstracts of the search results to select relevant papers for further screening. After removal of duplicates, the articles were obtained for screening/reading of the full text after which eligible papers were selected for inclusion in the systematic review. Discrepancies between the reviewers were resolved through consensus.

### Data extraction and management

Data extraction of the identified studies was done by using the CHARMS checklists (EA). Extracted data were checked (MAC) and disagreements were resolved by consensus (EA, MAC). In case of disagreement a third reviewer (KKG) was consulted. Studies were analysed qualitatively given the large variability of the studies included.

The following categories were extracted: authors, journal, year of publication, region or place where study was conducted, period of data collection, study design, inclusion and exclusion criteria, the sample size of the derivation cohort and/or the validation cohort, the gestational age at which women were enrolled into the study and the number of outcomes. Other information extracted were the number and types of predictors, the target population for whom the prediction model is intended for, the handling of missing data, the modeling method used, the model selection method, the handling of continuous data, the method used for internal validation and whether or not an external validation was done.

### Quality assessment

Quality of the studies was assessed using the CHARMS, STROBE and TRIPOD checklists and the National Institute of Health (NIH) [26] quality assessment tools for observational cohort and cross-sectional studies was independently assessed by two authors (EA, MAC). The NIH quality assessment tools focus on concepts that are key for critical appraisal of the internal validity of a study. The tool uses a 14-item checklist to assess the study design, inclusion criteria, outcome and variable description and collection and loss to follow up among others. Each item is scored as yes, no or other (not reported, not applicable or cannot determine). The tool also provides guidance on grading the studies as good, fair or poor. The studies were finally graded for risk of bias as”low” if risk of bias was unlikely, “moderate” if there were no essential flaws, but not all criteria had been satisfied and “high” if there were flaws in one or more important items. We adapted the tool and used 13 out of the 14 items, because one item, “for exposures that can vary in amount or level, did the study examine different levels of the exposure as related to the outcome (e.g., categories of exposure, or exposure measured as continuous variable)?” was not relevant to our review.

### Meta-analysis

We performed a meta-analysis on 22 of the studies with preeclampsia as outcome, using the MedCalc Statistical Software version 19.1.7 (MedCalc Software Ltd, Ostend, Belgium; https://www.medcalc.org; 2020). These 22 studies had fully reported the area under the curve with 95% confidence intervals. We used the random effects model.

## Results

**Fig 1:**
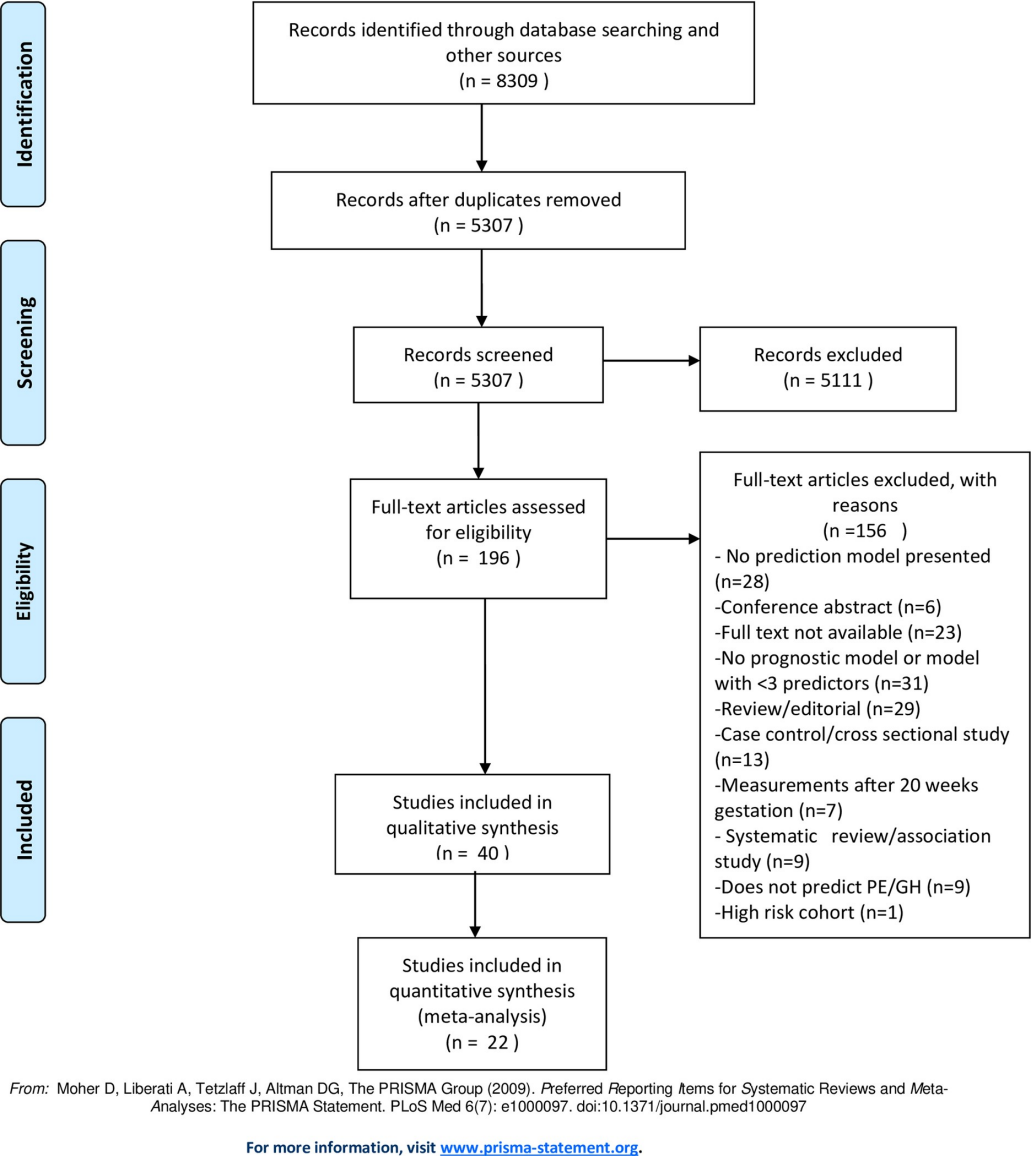
Flow diagram for inclusion and exclusion of relevant articles.

Fig 1 shows the flow diagram for inclusion and exclusion of relevant articles. The search yielded 8,309 papers. After removing 3,002 duplicates, 5307 papers were screened further for relevance and 196 papers selected for full text assessment. 156 articles were excluded based on reasons such as not presenting a prediction model, measurement of predictors done after 20 weeks of gestation and the prediction outcome not being preeclampsia or gestational hypertension. Finally 40 papers, published between 2000 and 2019, were selected for the review.

### Prediction models for gestational hypertension and pre-eclampsia

All forty studies included in this review were conducted between 2000 and 2019. Table 1 gives an overview of important parameters of the selected studies. The studies have been grouped in the following order: maternal characteristics only, maternal characteristics and uterine artery Doppler, maternal characteristics with biomarkers and maternal characteristics with biomarkers and uterine artery Doppler.

**Table 1:** Overview of prediction models.

**Study**	**Study design**	**Centre**	**Study population**	**Outcome**	**Women, n (outcome events; predictors)**	**Number of events per variable**
Mello et al, 2002 [14]	Prospective cohort	Single	Italian (Caucasian)	Preeclampsia	187 (47; 8))	5.9
Poon, et al, 2010 [34]	Prospective cohort	Single	United Kingdom (multi racial)	Early Preeclampsia, late preeclampsia, gestational hypertension.	8366 (165; 8)	20.6
Muto et al, 2016 [42]	Prospective cohort	Single	Japanese	Preeclampsia, gestational hypertension	1986 (50; 6)	8.3
Kuijk et al, 2014 [32]	Combined prospective and retrospective cohort	Multi centre	Dutch (multi racial)	Early onset preeclampsia	229(15; 5)	3
Poon et al, 2008 [35]	Prospective cohort	Single	United Kingdom (multi racial)	Preeclampsia, gestational hypertension	5193 (104; 5)	5
Benko et al, 2019 [53]	Prospective cohort	Multicentre	United Kingdom, Bulgaria, Spain (Multi racial)	Preeclampsia in twin pregnancies.	2219 (171;11)	15.5
Boutin et al, 2018 [58]	Prospective cohort	Single	Canadian (multi ethnic)	Preterm preeclampsia, all preeclampsia.	4612 (232;6)	38.7
Antwi et al, 2017 [47]	Prospective cohort	Multi centre	Ghanaian	Gestational hypertension	2529 (261; 6)	43.5
Becker Rolf, 2011 [49]	Retrospective cohort	Single	German (Caucasian)	Preeclampsia, preterm delivery, intrauterine fetal growth restriction, placental abruption, intrauterine fetal death, early neonatal fetal death (within first week of postnatal life)	15,855(172; 6)	28.7
North et al, 2011 [48]	Prospective cohort	Multi centre	United Kingdom, New Zealand, Ireland, Australia (multi racial)	Preeclampsia	3529(186; 13)	14.3
Sepulvelda-Martinez et al, 2019 [56]	Nested case control (Prospective cohort)	Single	Chilean	Preterm preeclampsia, term preeclampsia.	1756 (49; 7)	7
Myatt L. et al, 2012 [50]	Prospective cohort	Multi centre	American (multi racial)	Preeclampsia	2,394 (176; 7)	25.1
Goetzinger et al,2010 [51]	Retrospective cohort	Single	American (multi racial)	Preeclampsia	3716 (293; 5)	58.6
Odibo et al, 2011 [52]	Retrospective cohort	Single	American (multi racial)	Preeclampsia	452(42;6)	7
Kuijk et al. 2011 [19]	Prospective cohort	Multi centre	Dutch (multi racial)	Early onset preeclampsia	407 (28; 5)	5.6
Stamilio et al, 2000 [31]	Retrospective cohort	Single	American (multi racial)	Preeclampsia, Severe preeclampsia	1998 (49; 4)	12.2
Gabbay-Benziv et al, [23]	Prospective cohort	Multi centre	American (multi racial)	Preeclampsia	2433 (108; 5)	21.6
Allen et al, 2017 [44]	Prospective cohort	Single	United Kingdom (multi racial)	Preeclampsia, gestational hypertension, small-for-gestational age	1045 (56; 5)	11.2
Mello et al, 2001 [45]	Prospective cohort	Single	Italian (Caucasian)	Pregnancy induced hypertension	303 (76; 9)	8.4
Antwi et al, 2018 [60]	Prospective cohort	Multi centre	Ghananian	Gestational hypertension	373 (25;6)	4.1
Zhang et al, 2019 [57]	Prospective cohort	Single	Chinese	Early preeclampsia, late preeclampsi, small-for-gestational age baby.	3270 (43;8)	5.3
O’Gorman et al, 2016 [27]	Prospective cohort	Single	United Kingdom (multi racial)	Preterm Preeclampsia, term preeclampsia.	35,948 (1058; 15)	70.5
Paré et al, 2014 [28]	Prospective cohort	Multi centre	American (multi racial)	Preeclampsia, gestational hypertension, HELLP* syndrome, eclampsia	2,637 (431; 8)	29.6
Moon et al, 2015 [29]	Prospective cohort	Single	United Kingdom (multi racial)	Preeclampsia	1177(102;11)	9.3
Park et al, 2013 [30]	Prospective cohort	Multi centre	Australian (multi racial)	Early Preeclampsia, late preeclampsia, gestational hypertension.	3066 (83; 7)	11.9
Kenny et al, 2014 [33]	Prospective cohort	Multi center	New Zealand, Australia, United Kingdom, Ireland (multi racial)	Early onset preeclampsia, Preeclampsia	3529 (278; 5)	55.6
Poon et al, 2009 [21]	Prospective cohort	Single	United Kingdom (multi racial)	Early Preeclampsia, Late preeclampsia, gestational hypertension.	7797 (157; 8)	19.6
Herraiz et al, 2009 [36]	Prospective cohort	Single	Spanish (multi racial)	Early Preeclampsia, late preeclampsia	152 (20;4)	5
Di Lorenzo et al, 2012 [37]	Prospective cohort	Single	Italian (multi racial)	Early onset preeclampsia, late onset preeclampsia, overall Preeclampsia, gestational hypertension	2118 (preeclampsia(25), gestational hypertension (46); 8)	3.1
Goetzinger et al, 2014 [38]	Prospective cohort	Single	American (multi racial)	Preeclampsia	578(49; 6)	8.1
Crovetto et al, 2014 [39]	Nested case-control (Prospective cohort)	Single	Spanish (multi racial)	Early Preeclampsia, late preeclampsi	5759 (112; 10)	11.2
Gallo et al, 2016 [40]	Prospective cohort	Multi centre	United Kingdom (multi racial)	Preterm Preeclampsia, term preeclampsia.	7748 (268; 11)	24.4
Skrastad et al, 2015 [41]	Prospective cohort	Single	Norway	Preeclampsia, gestational hypertension	541 (21; 11)	1.9
Antonio et al, 2017 [43]	Prospective cohort	Single	Brazilian (multi racial)	Preeclampsia, gestational hypertension	617 (34; 4)	8.5
Parra-Cordero et al, 2013 [24]	Nested case-control (Prospective cohort)	Single	Chilean	Early onset Preeclampsia, late onset preeclampsia.	2619 (83; 4)	20.7
Myers et al, 2013 [20]	Prospective cohort	Multi centre	United Kingdom, New Zealand, Australia (multi racial)	Preterm preeclampsia	3529 (55; 7)	7.9
Baschat et al, 2014 [46]	Prospective cohort	Multi centre	American (multi racial)	Early onset preeclampsia, Preeclampsia	2441 (108; 5)	21.6
Scazzocchio, et al, 2017 [54]	Prospective cohort	Single	Spain	Early onset preeclampsia, late onset preeclampsia.	4203 (169; 7)	24.1
Wright et al, 2019 [55].	Prospective cohort	Multicentre	United Kingdom, Spain, Belgium, Italy, Greece	Early preeclampsia, pre-term preeclampsia. All preeclampsia.	61,174 (1770; 11)	160.9
Lobo et al, 2019 [59]	Prospective cohort	Single	Brazil (multi ethnic)	Preterm Preeclampsia, term preeclampsia	617 (34;8)	4.2
**Study**	**Predictors**	**Type of model**	**Internal validation**	**External validation**	**Calibration (p-value Hosmer-Lemeshow test or calibration plot)**	**Model performance: PPV, NPV, Sensitivity, Specificity,**
Mello et al, 2002 [14]	Maternal characteristics	Logistic regression	Yes	No	No	Yes
Poon, et al, 2010 [34]	Maternal characteristics	Logistic regression	Not stated	No	No	Yes
Muto et al, 2016 [42]	Maternal characteristics	Logistic regression	Not stated	No	No	Yes
Kuijk et al, 2014 [32]	Maternal characteristics	Logistic regression	Not applicable	Yes. Study externally validated a previously developed prediction model	Yes. Calibration plot and Hosmer-Lemeshow goodnesss -of-fit test.	Yes
Poon et al, 2008 [35]	Maternal characteristics	Logistic regression	Not stated	No	No	Yes
Benko et al, 2019	Maternal characteristics	Parametric survival model	Not stated	Yes	Yes	Yes
Boutin et al, 2018	maternal age, BMI, hypertension, chronic inflammatory disease, ovulation induction, in vitro fertilization	Proportional hazard model	Not stated	No	No	Yes
Antwi et al, 2017	Maternal weight, height, parity, diastolic blood pressure, history of gestational hypertension, family history of hypertension	Logistic regression	Bootstrapping	Yes	No	No
Becker Rolf, 2011 [49]	Maternal characteristics, uterine artery pulsatility index	Logistic regression	Not stated	Yes	No	No
North et al, 2011 [48]	Maternal characteristics, uterine artery pulsatility index	Logistic regression	Cross validation	No	Yes. Calibration plot	Yes
Sepulveda-Martinez et al, 2019	maternal characteristics, uterine artery pulsatility index	Logistic regression	Not stated	No	No	Yes
Myatt L. et al, 2012 [50]	Maternal characteristics, serum biomarkers	Logistic regression	Not stated	No	No	Yes
Goetzinger et al,2010 [51]	Maternal characteristics, serum biomarkers	Logistic regression	Not stated	No	No	Yes
Odibo et al, 2011 [52]	Maternal characteristics, serum biomarkers	Logistic regression	Not stated	No	No	Yes
Kuijk et al. 2011 [19]	Maternal characteristics, fasting blood glucose.	Logistic regression	Bootstrapping	No	Yes. Hosmer-Lemeshow goodnesss-of-fit test.	Yes
Stamilio et al, 2000 [31]	Maternal characteristics, serum biomarkers.	Logistic regression	Not stated	No	No	Yes
Gabbay-Benziv et al, [23]	Maternal characteristics, biomarkers.	Logistic regression	Not stated	No	No	Yes
Allen et al, 2017 [44]	Maternal characteristics, biomarkers.	Logistic regression	Not stated	No	No	Yes
Mello et al, 2001 [45]	Maternal characteristics, hematological and biochemical indices.	Logistic regression	Cross validation	No	No	Yes
Antwi et al, 2018 [47]	Maternal characteristics, serum biomarkers.	Logistic regression	Bootstrapping	Yes	Yes. Calibration plot	Yes
Zhang et al, 2019	BMI, ethicity, parity, history of preeclampsia, chronic hypertension, PAPP-A, PlGF		Not stated	No	No	Yes
O’Gorman et al, 2016 [27]	Maternal characteristics, serum biomarkers, uterine artery pulsatility index	Logistic regression	Not stated	No	No	Yes
Paré et al, 2014 [28]	Maternal characteristics, serum biomarkers, uterine artery pulsatility index	Logistic regression	Not stated	No	No	No
Moon et al, 2015 [29]	Maternal characteristics, serum biomarkers, uterine artery pulsatility index	Logistic regression	Not stated	No	No	Yes
Park et al, 2013 [30]	Maternal characteristics, serum biomarkers, uterine artery pulsatility index	Logistic regression	Not applicable because this study is an external validation of a previously developed prediction model	No	No	Yes
Kenny et al, 2014 [33]	Maternal characteristics, serum biomarkers, uterine artery pulsatility index	Logistic regression	Yes	No	No	Yes
Poon et al, 2009 [21]	Maternal characteristics, serum biomarkers, uterine artery pulsatility index	Logistic regression	Not stated	No	No	Yes
Herraiz et al, 2009 [36]	Maternal characteristics, serum biomarkers, uterine artery pulsatility index	Logistic regression	Not stated	Yes. Study externally validated a previously developed prediction model	No	Yes
Di Lorenzo et al, 2012 [37]	Maternal characteristics, serum biomarkers, uterine artery pulsatility index	Logistic regression	Not stated	No	No	Yes
Goetzinger et al, 2014 [38]	Maternal characteristics, serum biomarkers, uterine artery pulsatility index	Logistic regression	Not stated	Yes	Yes	Yes
Crovetto et al, 2014 [39]	Maternal characteristics, serum biomarkers, uterine artery pulsatility index	Logistic regression	Not stated	No	No	Yes
Gallo et al, 2016 [40]	Maternal characteristics, serum biomarkers, uterine artery pulsatility index	Logistic regression	Cross validation	No	No	Yes
Skrastad et al, 2015 [41]	Maternal characteristics, serum biomarkers, uterine artery pulsatility index	Logistic regression	Not stated	Yes. Study externally validated a previously developed prediction model	No	Yes
Antonio et al, 2017 [43]	Maternal characteristics, biomarkers, Uterine artery pulsatility index.	Logistic regression	Not stated	No	No	Yes
Parra-Cordero et al, 2013 [24]	Maternal characteristics, biomarkers, Uterine artery pulsatility index.	Logistic regression	Not stated	No	No	Yes
Myers et al, 2013 [20]	Maternal characteristics, biomarkers, Uterine artery pulsatility index.	Logistic regression	Cross validation	No	No	Yes
Baschat et al, 2014 [46]	Maternal characteristics, biomarkers, Uterine artery pulsatility index.	Logistic regression	Cross validation	No	No	Yes
Scazzocchio et al 2017	maternal characteristics, serum biomarkers, uterine artery pulsatility index	Logistic regression	Bootstrapping	Yes	Yes	Yes
Wright et al, 2019	maternal characteristics, MAP, Uterine artery pulsatility index, PlGF	Logistic regression	Not stated	Yes	Yes	Yes
Lobo et al, 2019	Maternal age, ethnicity, smoking status, MAP, Urerine artery pulsatility index, PlGF, PAPP-A	Fetal Medicine Foundation Algorithm	Not stated	Yes	No	Yes
**Study**	**Discrimination (AUC)**	**Prediction rule/score chart/nomogram**	**Handling of missing values**	**Model selection: Stepwise selection, Univariate p-values, No selection**	**Handling of continuous data: Kept linear, categorized, dichotomized**	
Mello et al, 2002 [14]	Yes; AUC (development) = 0.984; AUC (after external validation) = 0.892.	No	Not stated	Stepwise selection	Categorized	
Poon, et al, 2010 [34]	Yes; PE < 34 weeks: AUC = 0.794 (0.720 to 0.869);	Model formula with regression coefficients	Complete case analysis	Not stated	Kept linear	
	PE ≥ 34 weeks: AUC = 0.796 (0.761 to 0.830).					
Muto et al, 2016 [42]	No	Model formula with regression coefficients	Complete case analysis	Not stated	Categorized	
Kuijk et al, 2014 [32]	Yes; PE< 37 weeks: AUC = 62.4 (51.0 to 73.7). All PE:AUC = 61.4 (51.9 to 70.9)	Model formula with regression coefficients, score chart.	Regression imputation	Not stated	Categorized	
Poon et al, 2008 [35]	Yes; AUC = 0.852.	Model formula with regression coefficients	Complete case analysis	Not stated	Kept linear	
Benko et al, 2019	Yes; development cohort: AUC = 0.65 (0.60 to 0.69); validation cohort: AUC not stated.	Regression coefficients	Not stated	survival analysis	Not stated	
Boutin et al, 2018	AUC: 0.62 (0.58–0.66)	No	Complete case analysis	Univariate p-value	Not stated	
Antwi et al, 2017 [47]	Yes; development cohort: AUC = 0.70 (0.67 to 0.74); validation cohort: AUC = 0.68 (0.60 to 0.77).	Model formula with regression coefficients, score chart.	Multiple imputation	Stepwise backward selection	Kept linear	
Becker Rolf, 2011 [49]	No	Model formula with regression coefficients, algorithm.	Not stated	Not stated	Categorized	
North et al, 2011 [48]	Yes; AUC = 0.710 (0.706 to 0.714)	Model formula with regression coefficients	Imputation by expectation maximization method.	Stepwise backward selection	Kept linear, BMI categorized.	
Sepulveda-Martinez et al 2019	AUC: 0.890 (0.837–0.955)	Algorithm	Not stated	Stepwise backward selection	Not stated	
Myatt L. et al, 2012 [50]	Yes; AUC = 0.73 (0.69 to 0.77).	No	Complete case analysis	Stepwise backward selection	Kept linear	
Goetzinger et al,2010 [51]	Yes; AUC = 0.70 (0.65 to 0.72).	Model formula with regression coefficients	Complete case analysis	Stepwise backward selection	Categorized	
Odibo et al, 2011 [52]	Yes; AUC = 0.77 (0.63 to 0.81).	Model formula with regression coefficients	Complete case analysis	Stepwise backward selection	Kept linear	
Kuijk et al. 2011 [19]	Yes; AUC = 0.65 (0.56 to 0.74).	Model formula with regression coefficients	Single regression imputation	Not stated	Kept linear	
Stamilio et al, 2000 [31]	Yes; AUC = 0.75.	Model formula with regression coefficients	Complete case analysis	Stepwise backward selection	Categorized	
Gabbay-Benziv et al, [23]	Yes; 0.78 (0.72 to 0.85)	Prediction rule	Complete case analysis	Not stated	Categorized	
Allen et al, 2017 [44]	Yes; AUC = 0.81 (0.69 to 0.93)	Model formula with regression coefficients	Complete case analysis	Stepwise selection	Kept linear	
Mello et al, 2001 [45]	Yes; prediction at 16 weeks: AUC = 0.952 (0.895 to 1.000); prediction at 20 weeks: AUC = 0.851 (0.739 to 0.941)	Model formula with regression coefficients	Complete case analysis	Not stated	Categorized	
Antwi et al, 2018	AUC: 0.82 (0.74–0.89)	Model formula with regression coefficients	Complete case analysis	Stepwise backward selection	Kept linear	
Zhang et al, 2019	AUC for early PE: 0.90 (0.89–0.91); AUC for late PE: 0.82 (0.81–0.84)	PREDICTOR Algorithm	Complete case analysis	Not stated	Not stated	
O’Gorman et al, 2016 [27]	Yes; PE< 37 weeks: AUC = 0.907; PE ≥37 weeks: AUC = 0.796.	Model formula with regression coefficients	Complete case analysis	Stepwise backward selection	Kept linear	
Paré et al, 2014 [28]	No	Model formula with regression coefficients	Not stated	Stepwise backward selection	Kept linear	
Moon et al, 2015 [29]	Yes; Model nulliparous: AUC = 0.88 (0.80 to 0.94); Model multiparous: AUC = 0.84 (0.75 to 0.91).	Model formula with regression coefficients	Complete case analysis	Stepwise backward selection	Not stated	
Park et al, 2013 [30]	Yes; AUC = 0.926 (0.916–0.936).	Model formula with regression coefficients	Complete case analysis	Not stated	Kept linear	
Kenny et al, 2014 [33]	Yes; development cohort: AUC = 0.73(0.70 to 0.77); validation cohort: AUC = 0.68(0.63 to 0.74).	Model formula with regression coefficients	Imputation by expextation maximization method, complete case analysis for uterine artery pulsatility index	Stepwise backward selection	Kept linear	
Poon et al, 2009 [21]	No	model formula with regression coefficients	Complete case analysis	Not stated	Kept linear	
Herraiz et al, 2009 [36]	Yes; PE< 34 weeks: AUC = 0.779 (0.641 to 0.917); PE 34 weeks: AUC = 0.641 (0.481 to 0.801).	Model formula with regression coefficients	Not stated	Not applicable	Kept linear	
Di Lorenzo et al, 2012 [37]	Yes; AUC = 0.895	Model formula with regression coefficients	Complete case analysis	Step down procedure	Kept linear	
Goetzinger et al, 2014 [38]	Yes; development cohort: AUC = 0.80 (0.73 to 0.86); validation cohort: AUC = 0.78 (0.69 to 0.86).	Model formula with regression coefficients	Complete case analysis	Stepwise backward selection	Categorized	
Crovetto et al, 2014 [39]	Yes; AUC = 0.960 (0.919 to 0.999).	Model formula with regression coefficients	Not stated	Stepwise forward selection	Kept linear	
Gallo et al, 2016 [40]	Yes; PE<32 weeks: AUC = 0.995 (0.990 to 0.999); PE< 32 weeks: AUC = 0.930 (0.892 to 0.968); PE ≥ 37 weeks:AUC = 0.773 (0.771 to 0.805).	Model formula with regression coefficients	Complete case analysis	Not stated	Kept linear	
Skrastad et al, 2015 [41]	Yes; AUC (FMF*) = 0.77(0.67 to 0.87), AUC (PREDICTOR¥) = 0.74 (0.63–0.84)	Fetal Medicine Foundation algorithm	Complete case analysis	Not stated	Kept linear	
Antonio et al, 2017 [43]	Yes; PE <34 weeks: AUC = 0.946 (0.919 to 0.973); PE< 37 weeks: AUC = 0.870 (0.798 to 0.942); PE< 42 weeks: AUC = 0.857 (0.807 to0.907)	Model formula with regression coefficients	Complete case analysis	Not stated	Kept linear	
Parra-Cordero et al, 2013 [24]	ROC curve presented but AUC values not provided.	Model formula with regression coefficients	Complete case analysis	Not stated	Kept linear	
Myers et al, 2013 [20]	Yes; AUC = 0.84 (0.77 to 0.91)	No	Complete case analysis	Stepwise selection (forward selection followed by series of backward selection)	Age and blood pressure kept linear, BMI categorized	
Baschat et al, 2014 [46]	Yes; PE < 34 weeks: AUC = 0.83 (0.74 to 0.91); all PE: AUC = 0.82 (0.78 to 0.86).	Model formula with regression coefficients	Complete case analysis	Lasso logistic regression	Categorized	
Scazzocchio et al, 2017	Early onset PE AUC = 0.94 (95% CI, 0.88–0.99), late onset PE AUC = 0.72 (95% CI, 0.66–0.77)	Regression coefficients	Not stated	Not stated	Not stated	
Wright et al, 2019	Early PE:AUC = 0.95 (0.93–0.97); Pretem PE = 0.91 (0.89–0.91); All PE = 0.83 (0.81–0.84)	Algorithm	Not stated	Not stated	Not stated	
Lobo et al, 2019	Preterm PE AUC:0.94 (0.92–0.97); Term PE AUC: 0.87 (0.79–094)	FMF Algorithm	Complete case analysis	Not stated	Not stated	

Twelve studies were conducted in the United Kingdom, eight in the United States of America, four each in Australia, Spain and Italy and three in New Zealand. Two studies were done in the Netherlands, Ireland, Brazil, Chile and Ghana with one each in Japan, China, Germany, Norway, Bulgaria, Greece, Belgium and Canada.

Most of the studies were prospective cohort studies (33/40 = 82.5%), four were retrospective cohort studies (10%), three were nested-case control studies (7.5%) and one study combined a retrospective and prospective cohort design for data collection. The prediction models were derived through logistic regression or parametric survival modeling.

The gestational age at inclusion into the studies ranged between eight and twenty weeks. All the gestational ages were confirmed by ultrasound. The sample size for the studies ranged between 173 and 35,948. The events per variable in the studies ranged between 2.1 and 88.2.

**Fig 2:**
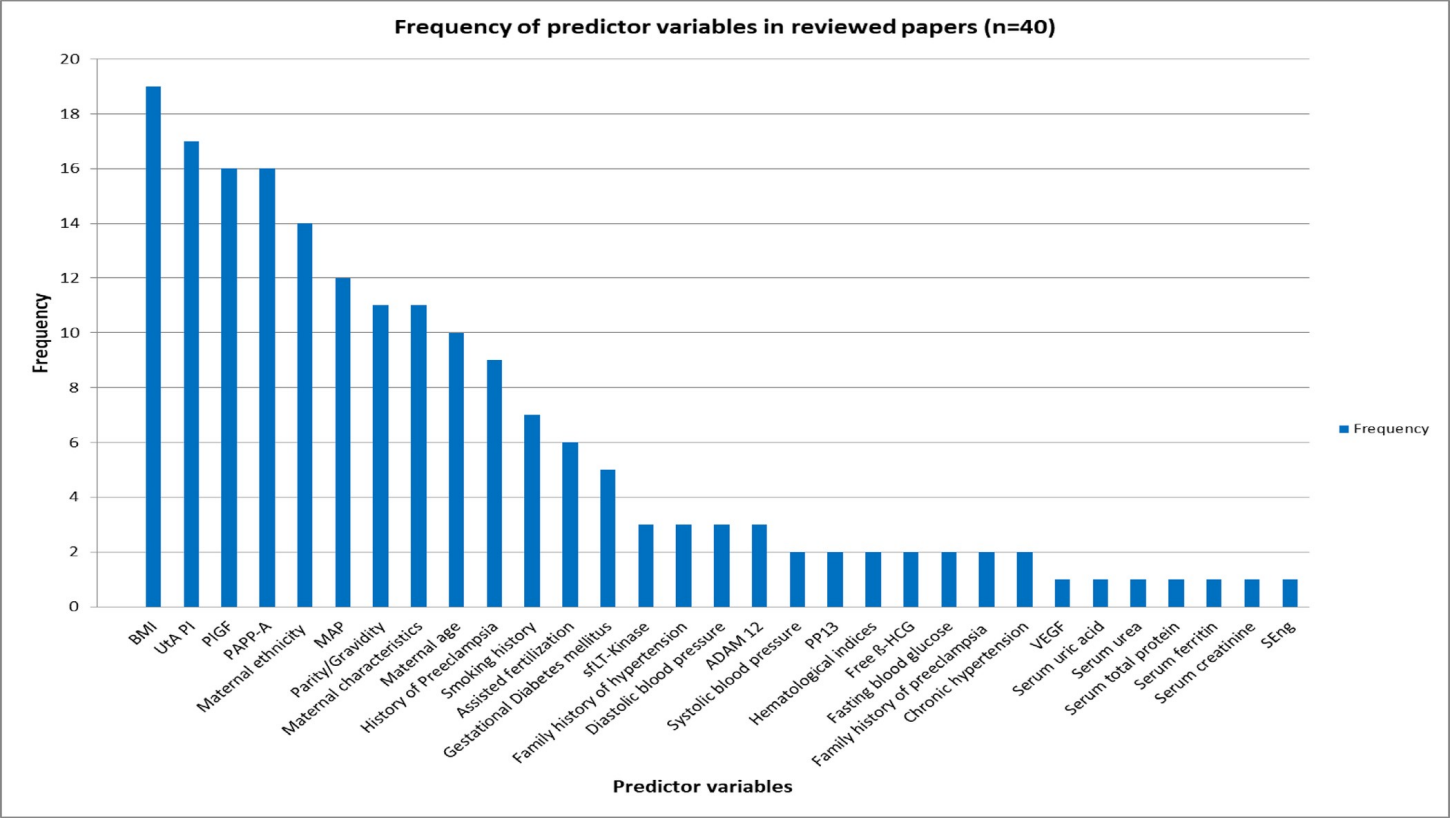
Frequency of predictor variables in the prediction models.

Seventy seven percent of all the prediction models combined biomarkers with maternal clinical characteristics. Body mass index (BMI) was the most frequently used predictor (19/40). Other maternal clinical predictors used in the models were first trimester systolic blood pressure and diastolic blood pressure, mean arterial pressure, maternal ethnicity, parity, previous history of preeclampsia, family history of hypertension, family history of preeclampsia, history of smoking and history of gestational diabetes mellitus. The following biomarkers were included: uterine artery pulsatility index (UtA PI, 17/40), pregnancy associated plasma protein-A (PAPP-A) (16/40) and placental growth factor (PlGF) (16/40). The following predictors were used less than ten times in the studies under review: free beta human chorionic gonadotropin (fß-HCG), alpha feto protein (AFP), soluble fms-like tyrosine kinase-1 (sFlt‐1), placental protein 13 (PP13), A disintegrin and metalloproteinase 12 (ADAM12), soluble endoglin (sEng) and vascular endothelial growth factor (VEGF). Fig 2 shows the frequency of predictor variables in the prediction models.

### Methodological quality of the studies to develop or validate prediction models using the CHARMS, STROBE and TRIPOD checklists

#### Source of data

All the studies indicated the type of study design used to obtain data for the prediction modeling. 37 were cohort studies whilst three were nested case-control studies.

#### Participants

All the studies indicated the participant eligibility and recruitment criteria, including the study location, number of centres and the inclusion and exclusion criteria.

#### Outcomes to be predicted

All the studies gave a standard definition for the outcome(s) to be predicted. Most of the studies had a single outcome while eleven studies had two or more outcomes.

#### Candidate predictors

All the studies defined and described the candidate predictors and the methods for their measurement. The timing of predictor measurements was also provided in all studies. Handling of predictors in the modeling process was described by 31 out of the 40 studies. Nine of the studies categorized continuous variables whilst 21 studies kept continuous variables linear.

#### Sample size

All studies provided the number of participants and the number of outcomes. Only nine of the studies explicitly estimated the sample size before the onset of the study. The number of outcomes in relation to the number of candidate predictors (events per variable) were deduced from the data and ranged between 2.1 and 88.2.

#### Missing data

The number of participants with any missing value for each predictor was not provided by the studies. Nine of the studies did not indicate how missing data were handled. Complete case analysis was used by 26 out of the 40 studies whilst five studies imputed missing data using the single regression imputation method [19,32], expectation maximization method [33,48] and multiple imputation [47].

#### Model development

All the studies selected candidate predictors for inclusion in the model through univariate analysis using a pre-determined p-value. Logistic regression and survival modelling were used to derive the prediction models. For selection of predictors during multivariable modeling, one study used the stepwise forward selection method, 14 studies used the stepwise backward selection method and two studies used stepwise selection without further specification. One study [46] applied the Lasso regression approach and another survival analysis whilst 21 studies did not state the method used for deriving the model.

#### Model performance

Discrimination of the prediction models, depicted by the c-statistic or the area under the receiver operating characteristic (ROC) curve was reported by 34 (85%) of the studies while calibration was reported by five (12.5%) studies. Classification measures were reported by 37 (92.5%) of the studies (Table 1).

### Model evaluation

#### Internal and external validation

Internal validation was reported by eleven out of 40 studies, using bootstrapping [19,47,54,60], cross validation [14,20,40,46,48], split sample [61] and back propagation of error method for artificial neural networks [45]. Nine out of the 40 prediction models were externally validated.

#### Risk of bias assessment

Risk of bias refers to the extent that flaws in the design, conduct, and analysis of the primary prediction modelling study lead to biased, often overly optimistic, estimates of predictive performance measures such as model calibration, discrimination, or (re)classification (usually due to overfitted models).

**Fig 3:**
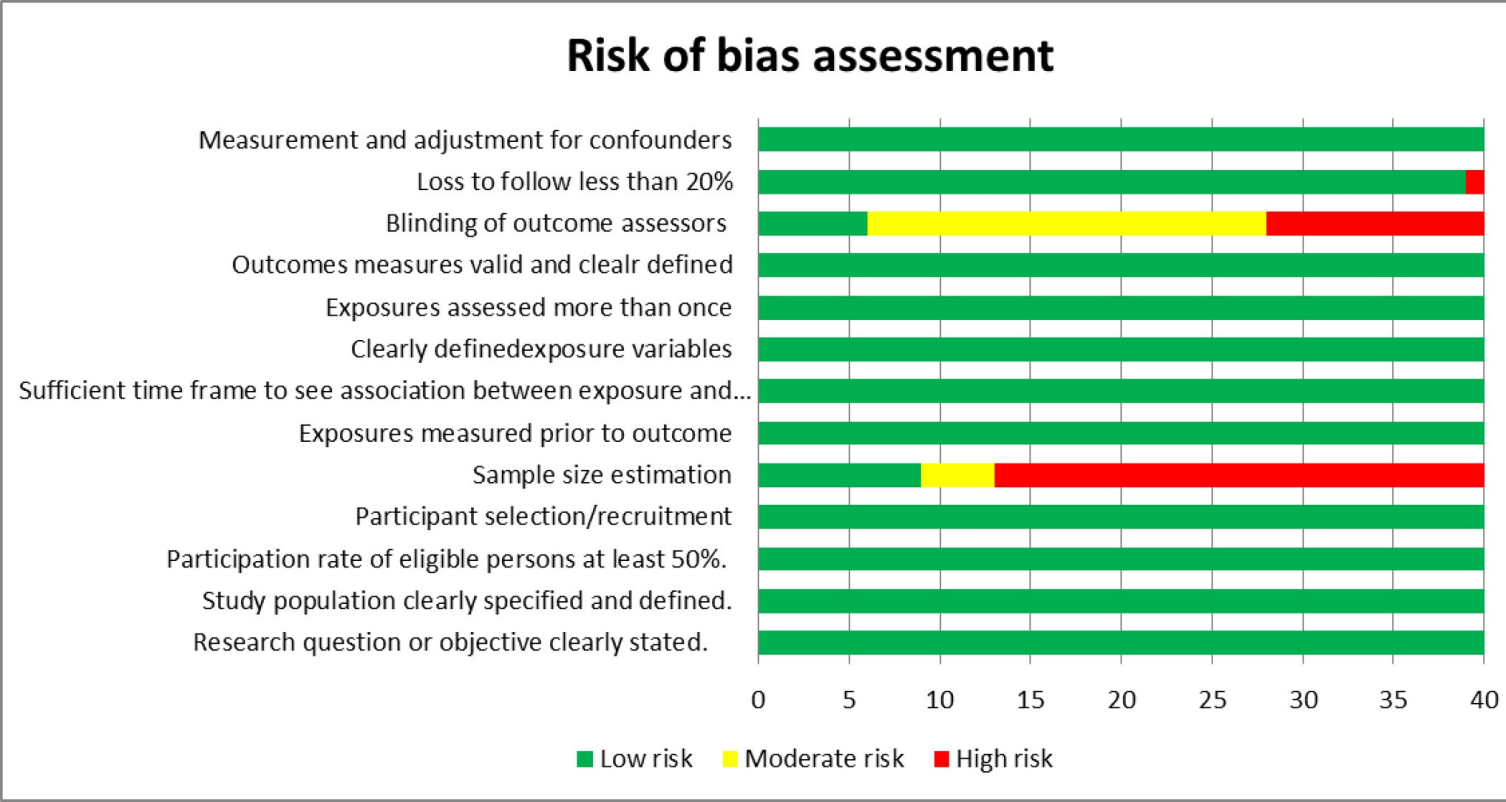
Risk of bias assessment of the prediction studies.

Fig 3 shows the risk of bias assessment of the studies. Most of the studies had a low risk of bias. The major source of bias related to sample size estimations, only stated in detail by nine out of 40 studies.

Details of the risk of bias assessment are presented in Table 2.

**Table 2:** Quality assessment of prediction model studies using the National Institute of Health criteria.

Study	Research question or objective in this paper clearly stated?	Study population clearly specified and defined?	Participation rate of eligible persons at least 50%?	Study subjects recruited from the same or similar populations (including the same time period)? Inclusion and exclusion criteria prespecified and applied uniformly to all participants?	Sample size justification, power description, or variance and effect estimates provided?	Exposure(s) of interest measured prior to the outcome(s) being measured?	Sufficient time frame to reasonably expect to see an association between exposure and outcome if it existed?	Exposure measures (independent variables) clearly defined, valid, reliable, and implemented consistently across all study participants?	Exposure(s) assessed more than once over time?	Outcome measures clearly defined, valid, reliable, and implemented consistently across all study participants?	Outcome assessors blinded to the exposure status of participants?	Loss to follow-up after baseline 20% or less?	Key potential confounding variables measured and adjusted statistically for their impact on the relationship between exposure(s) and outcome(s)?
G.Mello et al 2002	Yes	Yes	Yes (100%)	Yes	No	Yes	Yes	Yes	Yes	Yes	No	Yes (0)	Yes
Becker Rolf.	Yes	Yes	Yes (100%)	Yes	No	Yes	Yes	Yes	Yes	Yes	No	Yes (0)	Yes
Myatt L. et al.	Yes	Yes	Yes (100%)	Yes	Yes	Yes	Yes	Yes	Yes	Yes	Yes	Yes (1.9%)	Yes
Goetzinger et al	Yes	Yes	Yes (100%)	Yes	NR	Yes	Yes	Yes	Yes	Yes	Yes	Yes (7%)	Yes
Odibo et al.	Yes	Yes	Yes (94.8%)	Yes	NR	Yes	Yes	Yes	Yes	Yes	Cd	Yes (5.2%)	Yes
O’Gorman et al.	Yes	Yes	Yes (100%)	Yes	NR	Yes	Yes	Yes	Yes	Yes	CD	Yes (0)	Yes
Paré et al.	Yes	Yes	Yes (100%)	Yes	Yes	Yes	Yes	Yes	Yes	Yes	CD	No	Yes
Moon et al	Yes	Yes	Yes (100%)	Yes	CD	Yes	Yes	Yes	Yes	Yes	CD	Yes (1.9%)	Yes
Park et al.	Yes	Yes	Yes (98.1%)	Yes	No	Yes	Yes	Yes	Yes	Yes	CD	Yes (1.9%)	Yes
Van Kuijk et al.	Yes	Yes	Yes (100%)	Yes	No	Yes	Yes	Yes	Yes	Yes	No	Yes (0)	Yes
Stamilio et al.	Yes	Yes	Yes (100%)	Yes	No	Yes	Yes	Yes	Yes	Yes	CD	Yes (0)	Yes
Kenny et al.	Yes	Yes	Yes (99%)	Yes	No	Yes	Yes	Yes	Yes	Yes	CD	Yes (1%)	Yes
Poon, et al.	Yes	Yes	Yes (100%)	Yes	No	Yes	Yes	Yes	Yes	Yes	CD	Yes (0)	Yes
Poon et al	Yes		Yes (91.9%)	Yes	No	Yes	Yes	Yes	Yes	Yes	CD	Yes (8.1%)	Yes
Herraiz et al.	Yes	Yes	Yes (87.9%)	Yes	No	Yes	Yes	Yes	Yes	Yes	Yes	Yes (12.1%)	Yes
Di Lorenzo et al.	Yes	Yes	Yes (98%)	Yes	No	Yes	Yes	Yes	Yes	Yes	CD	Yes (2.4%)	Yes
Goetzinger et al.	Yes	Yes	Yes (98%)	Yes	No	Yes	Yes	Yes	Yes	Yes	CD	Yes (2%)	Yes
Crovetto et al.	Yes	Yes	Yes (100%)	Yes	No	Yes	Yes	Yes	Yes	Yes	CD	Yes (0)	Yes
Gallo et al.	Yes	Yes	Yes (100%)	Yes	No	Yes	Yes	Yes	Yes	Yes	CD	Yes (0)	Yes
Skrastad et al	Yes	Yes	Yes (96.6%)	Yes	No	Yes	Yes	Yes	Yes	Yes	Yes	Yes (3.4%)	Yes
Muto et al	Yes	Yes	Yes (100%)	Yes	No	Yes	Yes	Yes	Yes	Yes	CD	Yes (0)	Yes
Antonio et al.	Yes	Yes	87.6%	Yes	Yes	Yes	Yes	Yes	Yes	Yes	CD	Yes (12.4%)	Yes
Van Kuijk et al.	Yes	Yes	Yes (100%)	Yes	No	Yes	Yes	Yes	Yes	Yes	CD	Yes (0)	Yes
Gabbay-Benziv et al.	Yes	Yes	Yes (100%)	Yes	No	Yes	Yes	Yes	Yes	Yes	CD	Yes (0)	Yes
Poon et al.	Yes	Yes	Yes (92.9%)	Yes	No	Yes	Yes	Yes	Yes	Yes	CD	Yes (7.1%)	Yes
Allen et al.	Yes	Yes	Yes (83.6%)	Yes	Yes	Yes	Yes	Yes	Yes	Yes	CD	Yes (16.4%)	Yes
Parra-Cordero et al.	Yes	Yes	Yes (100%)	Yes	Yes	Yes	Yes	Yes	Yes	Yes	CD	Yes (0)	Yes
Myers et al.	Yes	Yes	Yes (99%)	Yes	Yes	Yes	Yes	Yes	Yes	Yes	CD	Yes (1%)	Yes
Mello et al.	Yes	Yes	Yes (100%)	Yes	No	Yes	Yes	Yes	Yes	Yes	Yes	Yes (0)	Yes
Baschat et al.	Yes	Yes	Yes (100%)	Yes	No	Yes	Yes	Yes	Yes	Yes	CD	Yes (0)	Yes
Antwi et al.	Yes	Yes	Yes (100%)	Yes	Yes	Yes	Yes	Yes	Yes	Yes	No	Yes (0)	Yes
North et al.	Yes	Yes	Yes (94.8%)	Yes	Yes	Yes	Yes	Yes	Yes	Yes	No	Yes (5.2%)	Yes
Antwi et al, 2018	Yes	Yes	Yes (100%)	Yes	No	Yes	Yes	Yes	Yes	Yes	No	Yes	Yes
Benko et al	Yes	Yes	Yes	Yes	No	Yes	Yes	Yes	Yes	Yes	No	Yes	Yes
Scazzocchio et al	Yes	Yes	Yes (100%)	Yes	No	Yes	Yes	Yes	Yes	Yes	No	Yes	Yes
Sepulvelda-Martinez	Yes	Yes	Yes	Yes	No	Yes	Yes	Yes	Yes	Yes	No	Yes	Yes
Wright et al	Yes	Yes	Yes	Yes	No	Yes	Yes	Yes	Yes	Yes	No	Yes	Yes
Zhang et al	Yes	Yes	Yes	Yes	No	Yes	Yes	Yes	Yes	Yes	No	Yes	Yes
Boutin et al	Yes	Yes	Yes	Yes	No	Yes	Yes	Yes	Yes	Yes	No	Yes	Yes
Lobo et al	Yes	Yes	Yes	Yes	No	Yes	Yes	Yes	Yes	Yes	No	Yes	Yes

CD- Could not be determined; NR- Not reported.

#### Prediction models applicable in low and middle income settings

Apart from two models each from Brazil and Chile, both Upper middle income countries in Latin America, and two models from Ghana, all the other models in the literature that met our inclusion criteria were developed in high income countries of Europe, Japan, Australia, New Zealand, China, Canada and the United States of America.

#### Meta-analysis

**Fig 4:**
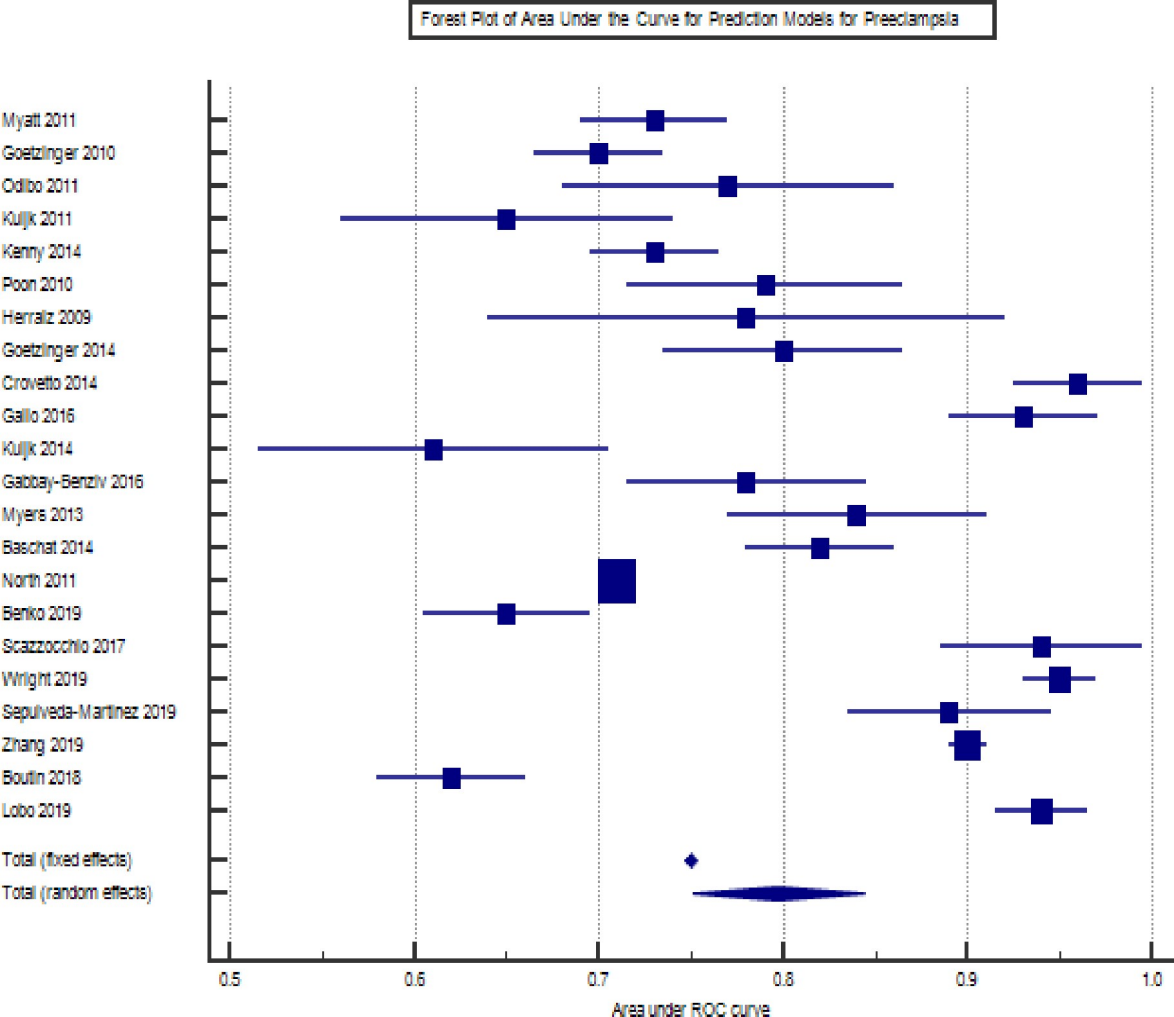
Forest plot of prediction models for preeclampsia.

The forest plot of the meta-analysis of the prediction models for preeclampsia is presented in Fig 4. The I^2^ was 99%. Overall area under the curve was 0.79 (0.75–0.84).

## Discussion

We set out to review the evidence in the published literature on the performance of multivariate prediction models for gestational hypertension and preeclampsia to assess the effectiveness of prediction models in identifying pregnant women at risk for gestational hypertension and preeclampsia. The specific objectives of this study were to identify prediction models for gestational hypertension and preeclampsia in the literature, assess the methodological quality of the prediction modeling studies by applying the CHARMS checklist and identify prediction models that can be applied in low and middle income country settings.

### Prediction models for gestational hypertension and preeclampsia

Our study identified 40 prediction models for gestational hypertension and preeclampsia, most of which had been developed and validated in high-income countries in Europe, Australia and the USA. Only two of such studies had been conducted in a low and middle income country setting. Most of the prediction models were developed in single centres but a few had been developed using data from multiple centres in one or more countries.

### Methodological quality of prediction modeling studies

The STROBE (Strengthening the reporting of observational studies in epidemiology), TRIPOD (Transparent reporting of a multivariable prediction model for individual prognosis or diagnosis) and the CHARMS checklists have outlined steps for developing and validating prediction models. The CHARMS checklist in particular provides guidance as to the items to extract when conducting a systematic review of prediction studies. An assessment of the methods used in model development in the studies evaluated in this review showed gaps in application of recommendations in the CHARMS, TRIPOD and STROBE checklists. The following domains of the CHARMS checklist were not adequately addressed in most of the studies: the source of data, study participants, outcome(s) to be predicted, candidate predictors, sample size, missing data, model development, model performance, model evaluation, results, interpretation and discussion. For example continuous predictors were dichotomized in some of the studies despite evidence and recommendations to the contrary [62–65]. Bias in predictor selection is known to occur when continuous predictors are categorized. Again, categorizing continuous variables assumes that there is a stepwise change in risk from one cut-off point to another. Bodnar et al [66] have demonstrated a dose-dependent relationship between pre-pregnancy BMI and the risk of preeclampsia. As BMI increases, so does the risk of preeclampsia. Therefore categorizing the predictor variable makes the functional relationship between the continuous variable (predictor) and the outcome variable linear, hence nonlinear transformations such as restricted cubic splines or fractional polynomials cannot be applied [62,67,68].

To prevent overestimation of risks by prediction models, it is recommended that the number of outcomes in relation to the number of predictors (events-per-variable) should be at least ten to one [69,70]. This requires an adequate sample size that ensures that there are enough outcomes in the study. Hence sample size estimation is an important methodological consideration so that at the onset of the study an adequate events-per-variable can be assured and thereby prevent overestimation of the predictive performance of the models (overfitting). Unfortunately, most of the studies under review did not report on sample size estimation. An adequate sample size also minimizes predictor selection bias. Predictor selection bias tends to be greater in smaller datasets when the events-per-variable ratio is small, especially when there are weak predictors in the dataset [16].

Information on missing data should be reported as part of the results of the studies. This includes the number of participants with any missing value (including values for both predictors and outcomes), number of participants with missing data for each predictor and how the missing data were handled, for example by complete case analysis, imputation or other methods. Information about missing data gives an idea as to the extent of bias, dependent on the reasons for the missing data. Where data were not missing completely at random, the prediction estimates are likely to be biased [64,71–75]. Missing data are seldom missing completely at random and may often be related to other observed participant data. Consequently, participants with completely observed data are likely to be different from those with missing data. Complete-case analysis which was the commonest method used to handle missing data in most studies deletes participants with a missing value from the analysis, thereby resulting in loss of information from a subset of the study population. This may result in over or under estimation of the predictive effect and reduced performance in an external population.

Prediction model performance is one of the important domains to be in the reported on [71]. Model performance indicators include calibration, discrimination and classification. It is recommended that discrimination and calibration should always be reported for prediction models. Discrimination indicates how well the prediction model distinguishes between two outcomes such as disease or non-disease and is assessed using the c-statistic or the area-under-the-curve (AUC) of a receiver operating characteristic curve [76–78]. The AUC ranges from 0.5 to 1 and represents the prediction model’s ability to correctly classify a randomly selected individual as being from one of two hypothetical populations [78–81]. An AUC value of 1.0 is considered perfect, 0.9–0.99 excellent, 0.8–0.89 good, 0.7–0.79 fair and 0.51–0.69 poor. An AUC of 0.5 is considered non-informative. The AUC in the studies under review ranged between 0.65 and 0.98. Apart from the study by Kuijk et al [19] which had an AUC of 0.65, all the other studies reported AUC greater than or equal to 0.70, indicating good to excellent discrimination. Calibration refers to how well the predicted risks compare to the observed outcomes. Usually this is evaluated in a calibration plot by graphically plotting observed against predicted event rates [16,67,82]. Calibration plots may be supplemented by the Hosmer-Lemeshow test, which is a formal statistical test to determine whether calibration is adequate. Unfortunately most of the studies under review did not report the calibration plot. This shortcoming leaves room for uncertainty in applying the model in clinical practice because one cannot determine the probability range within which the model works well. Both discrimination and calibration are essential in determining model performance.

Prediction model evaluation can be undertaken by internal validation (using the same dataset as that used to develop the model) and external validation (using a different dataset to that used in developing the model). The external dataset should be collected using the same predictor and outcome definitions and measurements. Again most of the studies did not report whether or not internal validation had been performed thus breaching an important methodological consideration. Most of the studies did not follow the guidelines in the TRIPOD, STROBE and CHARMS checklists. A possible explanation may be that some of studies were conducted prior to the development of these guidelines so the investigators may not have had the benefit of these methodological guidelines.

### Prediction models applicable in low and middle income settings

Only five of the studies had been conducted in a low-and-middle income country setting. Given contextual differences between high and low-and-middle income countries, many of the prediction models under review which have been developed in high income countries at present may not be applicable in most low-and-middle income countries. This is because these prediction models included biomarkers and uterine artery pulsatility index as predictors in addition to maternal clinical characteristics [20,21,23,24,27,28,30,36–41,44,46,48–52,61,83]. At present uterine Doppler measurement and serum biomarker assays are not widely available in many low-and-middle income countries. Therefore prediction models using biomarkers and uterine artery pulsatility index may not be routinely applied in these settings.

Generally, prediction models developed in one setting have to be externally validated in new populations to assess their performance before applying them in clinical decision-making. The model intercept and the regression coefficients often have to be updated to fit the new context or population to which the prediction model is being applied to. Thus prediction models developed elsewhere may be updated for use in other settings provided the predictors and outcome are the same. In situations where a prediction model includes variables which cannot be measured in the setting where the model is to be applied, that model cannot be used in that population. Consequently most prediction models developed in high income countries and including variables like serum biomarkers and uterine artery pulsatility index are at present not applicable in most low-and-middle income countries where the burden of hypertensive disorders of pregnancy is greater. Presently prediction models using maternal clinical characteristics, and which give optimum predictions can be externally validated and applied in low resource settings.

## Conclusion

Most of the studies evaluated did not completely follow the CHARMS, TRIPOD and STROBE guidelines in prediction model development and reporting. Adherence to these guidelines will improve prediction modelling studies and subsequent application of prediction models in clinical practice. Prediction models using maternal characteristics, with good discrimination and calibration, should be externally validated for use in low and middle income countries where biomarker assays are not routinely available.

## Supporting information

S1 Data Search strategy for PubMed. (DOCX)

S2 Data Standard error of area under the curve used to build the forest plot. (DOCX)
