# Supplementary material for: Systematic review of prediction models for gestational hypertension and preeclampsia
Source: PLoS One. 2020 Apr 21;15(4):e0230955. doi: 10.1371/journal.pone.0230955 (PMC7173928; doi:10.1371/journal.pone.0230955)
Supplement: S1 Data — (DOCX) [file pone.0230955.s001.docx]

S1. Search strategy for PubMed

Search **((((((((((((risk) OR risks) OR probability) OR probabilities) OR causality) OR causation) OR enabling factor) OR enabling factors) OR predisposing factor) OR predisposing factors)) AND ((((((((((((((prediction models) OR prediction model) OR prediction) OR predictive) OR forecasting) OR probability learning) OR decision support technique) OR decision support techniques) OR decision support model) OR decision support models) OR decision analysis) OR decision analyses) OR clinical prediction rule) OR clinical prediction rules)) AND ((((((((((((((gestational hypertension) OR pregnancy induced hypertension) OR pregnancy transient hypertension) OR maternal hypertension) OR preeclampsia) OR pre-eclampsia) OR pre-eclamptic) OR pregnancy toxemia) OR pregnancy toxemias) OR edema proteinuria hypertension gestosis) OR eph complex) OR eph toxemia) OR eph gestosis) OR proteinuria edema gestosis)**
