# Supplementary material for: Systematic review of prediction models for gestational hypertension and preeclampsia
Source: PLoS One. 2020 Apr 21;15(4):e0230955. doi: 10.1371/journal.pone.0230955 (PMC7173928; doi:10.1371/journal.pone.0230955)
Supplement: S2 Data — (DOCX) [file pone.0230955.s002.docx]

S2. Standard error of area under the curve used to build the forest plot.

| Variable for studies | | Study | | | | | | |
| --- | --- | --- | --- | --- | --- | --- | --- | --- |
| Variable for Area under ROC curve (AUC) | | AUC | | | | | | |
| Variable for Standard Error of AUC | | Standard error | | | | | | |
| Study | ROC Area | | Standard Error | 95% CI | z | P | Weight (%) | |
|  |  |  |  |  |  |  | Fixed | Random |
| Myatt 2011 | 0.730 | | 0.0204 | 0.690 to 0.770 |  |  | 0.72 | 4.73 |
| Goetzinger 2010 | 0.700 | | 0.0179 | 0.665 to 0.735 |  |  | 0.94 | 4.77 |
| Odibo 2011 | 0.770 | | 0.0459 | 0.680 to 0.860 |  |  | 0.14 | 4.14 |
| Kuijk 2011 | 0.650 | | 0.0459 | 0.560 to 0.740 |  |  | 0.14 | 4.14 |
| Kenny 2014 | 0.730 | | 0.0179 | 0.695 to 0.765 |  |  | 0.94 | 4.77 |
| Poon 2010 | 0.790 | | 0.0383 | 0.715 to 0.865 |  |  | 0.20 | 4.35 |
| Herraiz 2009 | 0.780 | | 0.0714 | 0.640 to 0.920 |  |  | 0.059 | 3.40 |
| Goetzinger 2014 | 0.800 | | 0.0332 | 0.735 to 0.865 |  |  | 0.27 | 4.47 |
| Crovetto 2014 | 0.960 | | 0.0179 | 0.925 to 0.995 |  |  | 0.94 | 4.77 |
| Gallo 2016 | 0.930 | | 0.0204 | 0.890 to 0.970 |  |  | 0.72 | 4.73 |
| Kuijk 2014 | 0.610 | | 0.0485 | 0.515 to 0.705 |  |  | 0.13 | 4.07 |
| Gabbay-Benziv 2016 | 0.780 | | 0.0332 | 0.715 to 0.845 |  |  | 0.27 | 4.47 |
| Myers 2013 | 0.840 | | 0.0357 | 0.770 to 0.910 |  |  | 0.24 | 4.41 |
| Baschat 2014 | 0.820 | | 0.0204 | 0.780 to 0.860 |  |  | 0.72 | 4.73 |
| North 2011 | 0.710 | | 0.00200 | 0.706 to 0.714 |  |  | 75.11 | 4.90 |
| Benko 2019 | 0.650 | | 0.0229 | 0.605 to 0.695 |  |  | 0.57 | 4.69 |
| Scazzocchio 2017 | 0.940 | | 0.0280 | 0.885 to 0.995 |  |  | 0.38 | 4.59 |
| Wright 2019 | 0.950 | | 0.0100 | 0.930 to 0.970 |  |  | 3.00 | 4.86 |
| Sepulveda-Martinez 2019 | 0.890 | | 0.0281 | 0.835 to 0.945 |  |  | 0.38 | 4.59 |
| Zhang 2019 | 0.900 | | 0.00510 | 0.890 to 0.910 |  |  | 11.55 | 4.89 |
| Boutin 2018 | 0.620 | | 0.0204 | 0.580 to 0.660 |  |  | 0.72 | 4.73 |
| Lobo 2019 | 0.940 | | 0.0128 | 0.915 to 0.965 |  |  | 1.83 | 4.83 |
| Total (fixed effects) | 0.750 | | 0.00173 | 0.746 to 0.753 | 432.530 | <0.001 | 100.00 | 100.00 |
| Total (random effects) | 0.797 | | 0.0238 | 0.751 to 0.844 | 33.442 | <0.001 | 100.00 | 100.00 |
